# Risk factor analysis of autoimmune hemolytic anemia after allogeneic hematopoietic stem cell transplantation in children

**DOI:** 10.1097/MD.0000000000005396

**Published:** 2016-11-18

**Authors:** Tsung-Yen Chang, Tang-Her Jaing, Yu-Chuan Wen, I-Anne Huang, Shih-Hsiang Chen, Pei-Kwei Tsay

**Affiliations:** a Divisions of Hematology/Oncology; b Division of Pediatric General Medicine, Department of Pediatrics; c Department of Nursing, Chang Gung Children's Hospital; d Department of Public Health and Center of Biostatistics, College of Medicine, Chang Gung University, Linkou, Taoyuan, Taiwan.

**Keywords:** risk factor, autoimmune hemolytic anemia, allogeneic hematopoietic stem cell transplantation, children

## Abstract

Autoimmune hemolytic anemia (AIHA) is a clinically relevant complication after allogeneic hematopoietic stem cell transplantation (HSCT). Currently, there is no established consensus regarding the optimal therapeutic approach. Whether AIHA contributes to increased mortality is still somewhat controversial.

We investigated the incidence, risk factors, and outcome of post-transplant AIHA in 265 consecutive pediatric patients undergoing allo-HSCT over a 17-year period. Onset of AIHA was calculated from the first documented detection of AIHA by either clinical symptoms or positive direct agglutinin test. Resolution of AIHA was defined as normalization of hemoglobin and biochemical markers of hemolysis with sustained transfusion independence.

We identified 15 cases of AIHA after allo-HSCT (incidence rate, 6%). Ten (67%) of these patients had a positive direct antiglobulin test. Data were obtained for 9 boys and 6 girls after a median follow-up of 53 months (range 4–102). The median age was 5.1 years (range 0.5–15.4) at the time of HSCT and the median time to emergence was 149 days (range 42–273). No significant risk factor for post-transplant AIHA has emerged from our data to date. In the majority (14 of 15; 93%) of AIHA patients, multiple agents for treatment were required, with 12 of 15 (80%) patients achieving complete resolution of AIHA. No splenectomy was performed in any of our patients.

For various reasons, post-transplantation AIHA poses an extraordinary challenge to transplant physicians. Despite the advancements in diagnostic tools, therapeutic challenges remain due to the myriad interacting pathways in AIHA.

## 1 Introduction

The development of autoimmune diseases is an increasingly recognized complication after hematopoietic stem cell transplantation (HSCT).^[1,2]^ Autoimmune hemolytic anemia (AIHA) is frequently resistant to treatment and confers decreased overall survival.^[3]^ The clinical significance of these risk factors for developing AIHA remains uncertain, as they have not been consistently reported among studies. Traditional therapies in children with AIHA include immune modulators such as steroids or intravenous immunoglobulin (IVIG). For a fraction of children, these first-line agents are ineffective, or clinically stable status can be maintained only by continuing steroids at the risk of long-term side effects.

Reasonable estimates of the incidence of AIHA after HSCT are between 2% and 6%, affecting both adult and pediatric patients.^[4,5]^ Higher incidences of up to 15% to 20% have been reported,^[6]^ albeit in specific clinical settings and with less conventional conditioning regimens. Transfusing AIHA patient is a challenge to the immunohematologist as it is encountered with difficulties in ABO grouping and cross matching requiring specialized serological tests such as alloadsorption or autoadsorption.^[7]^ To better understand the risk factors, prognosis, and management of post-HSCT AIHA, we carried out a retrospective analysis of 265 allogeneic HSCTs performed at Chang Gung Children's Hospital between 1998 and 2015.

## 2 Materials and methods

### 2.1 Patients

Between April 1998 and June 2015, 265 allogeneic HSCTs carried out at Chang Gung Children's Hospital were recruited in the longitudinal study. In patients who received >1 HSCT, each transplant was counted as a separate event if the graft was received from a different donor. The median follow-up period after HSCT was 86 months (range 1–206 months). The retrospective review was approved by the local research ethics committee and written informed consent was obtained from all parents or guardians. Patients with an HLA-matched sibling donor received the graft from the sibling; otherwise, a suitable unrelated donor was identified.

### 2.2 Transplantations protocols

The selection of conditioning regimen and source of hematopoietic stem cells were based on hematological diagnosis, donor availability, and clinical state of the patient. Preparation of cord blood units for infusion by a bedside thaw without centrifugation to minimize undesirable cell loss is conducted in our center. Graft-versus-host disease (GVHD) prophylaxis comprised short-course methotrexate or methylprednisolone and cyclosporine administered for a minimum of 6 months and tapered thereafter. Chimerism analysis was performed routinely on the day of neutrophil engraftment, days 42 and 100, and then 6 months after HSCT using single nucleotide polymorphism analysis. Red cells and platelets were transfused to maintain hemoglobin level and platelet count >80 g/L and 20 × 10^9^/L, respectively. The conditioning regimen and transplantation characteristics of our patients are shown in Table 1.

**Table 1:**
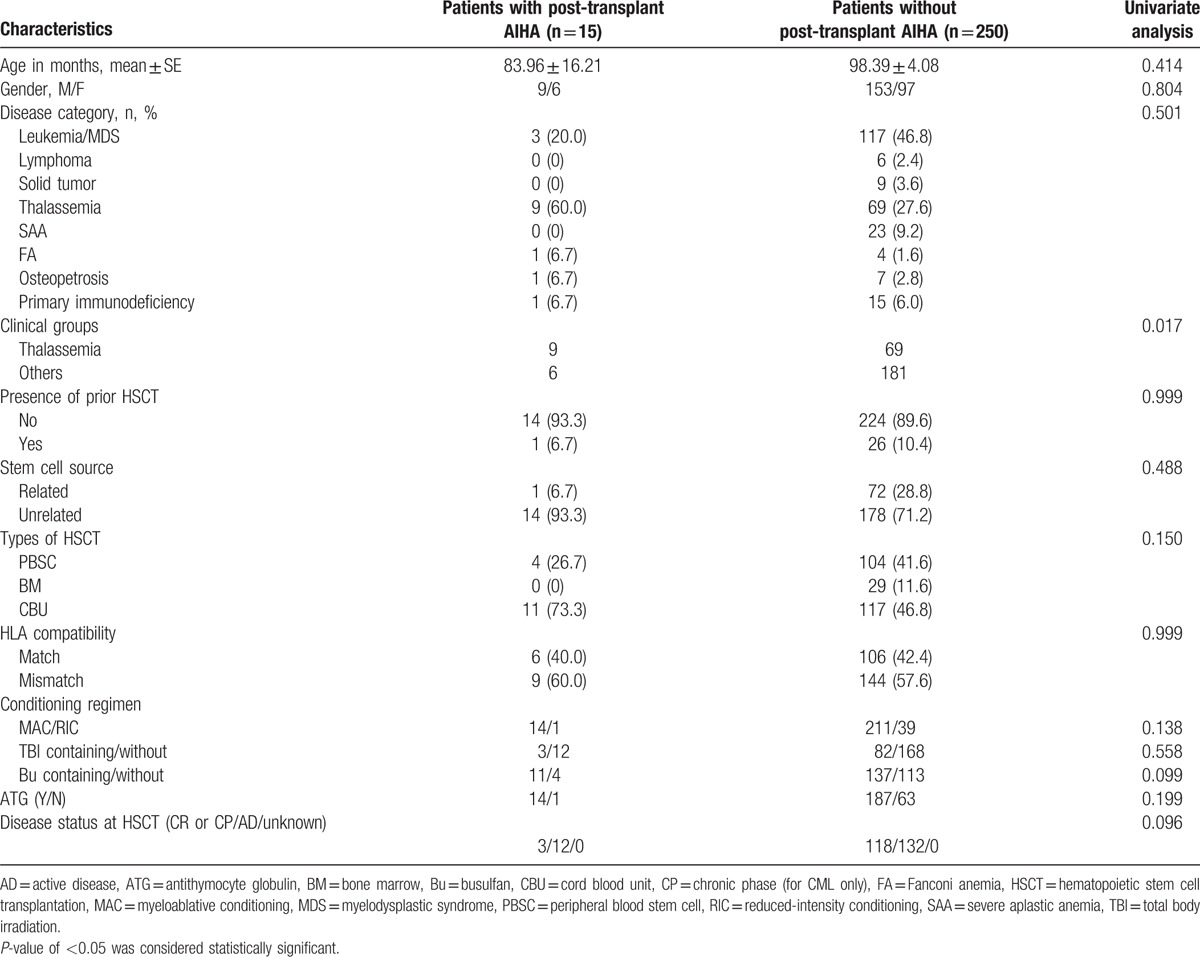
Comparison between patients with and without AIHA by univariate analysis.

### 2.3 Diagnosis and treatment of AIHA

We identified all children treated for auto-immune cytopenias at our institution through a comprehensive search of the hospital data warehouse cross-referenced with our HSCT program and research databases. Diagnosis is primarily made by 2 criteria: symptoms of hemolysis and anemia, and positive direct antiglobulin test (DAT, Coombs test).^[8,9]^ Response was defined as a rise in hemoglobin by at least 15 g/L and to a level of 100 g/L or greater. Other infections and hematological malignancies were ruled out by appropriate diagnostic procedures. Resolution of AIHA was defined as normalization of hemoglobin and biochemical markers of hemolysis and independence from additional treatment.

Criteria for rituximab therapy for post-transplant AIHA were evidence of hemolysis (anemia, raised reticulocyte count, elevated indirect bilirubin, and lactate dehydrogenase), positive DAT and failure of first-line therapy. Rituximab (MABTHERA, Roche, Basel, Switzerland) were given intravenously at a dose of 375 mg/m^2^ once per week, if clinically indicated. The study was approved by the independent institutional review board of the study center.

### 2.4 Laboratory evaluation

The DAT was conducted to detect the presence of IgG or IgM antibodies and complement on the patient's red blood cells (Immucor, Norcross, GA). If initial testing was positive, further testing was conducted to determine if the reaction was the result of IgG and/or complement specifically C3. If IgG antibodies were present, an eluate was prepared and tested to determine antibody specificity.

### 2.5 Statistical analysis

The primary endpoint was the onset of AIHA. A Mann–Whitney *U*-test was used to compare patients with or without AIHA. Risk factors for developing AIHA were evaluated by univariate and multivariate analyses using the Cox regression model. Categorical variables were summarized by frequency and percentages, and their comparison was carried out by either chi-square test or Fisher's exact test. Values of *P* < 0.05 were considered significant. Other factors considered as potential confounders in the regression analyses were recipient gender, primary hematological disease, HSCT type (unrelated versus sibling), source of stem cells (peripheral blood versus bone marrow versus cord), conditioning regimen (myeloablative versus reduced intensity), human leukocyte antigen (HLA) mismatch between the donor and the recipient, gender mismatch between the donor and the recipient, recipient cytomegalovirus (CMV) status, and concurrent chronic GVHD. The secondary endpoint was mortality. SPSS statistics for Windows (SPPS Inc., Chicago, IL), version 18.0, was used for these analyses.

## 3 Results

### 3.1 Clinical characteristics

Records of 265 HSCT patients were analyzed for evidence of AIHA, but only 15 of these cases exhibited clinically significant hemolysis to be classified as AIHA, resulting in an overall incidence of 6%. Patients with thalassemia had a higher rate of AIHA than patients other than thalassemia (11.5% vs 3.2%; *P* = 0.017). Clinical characteristics of the AIHA cases are summarized in Table 2. The range to onset of AIHA was observed to be between 42 and 273 days after HSCT, whereas no serious concurrent infection was recorded. At the onset of AIHA, all patients were receiving immunosuppressive therapies, either as GVHD treatment or prophylaxis. All patients were required to intensify transfusional support by reducing the intervals between transfusions.

**Table 2:**
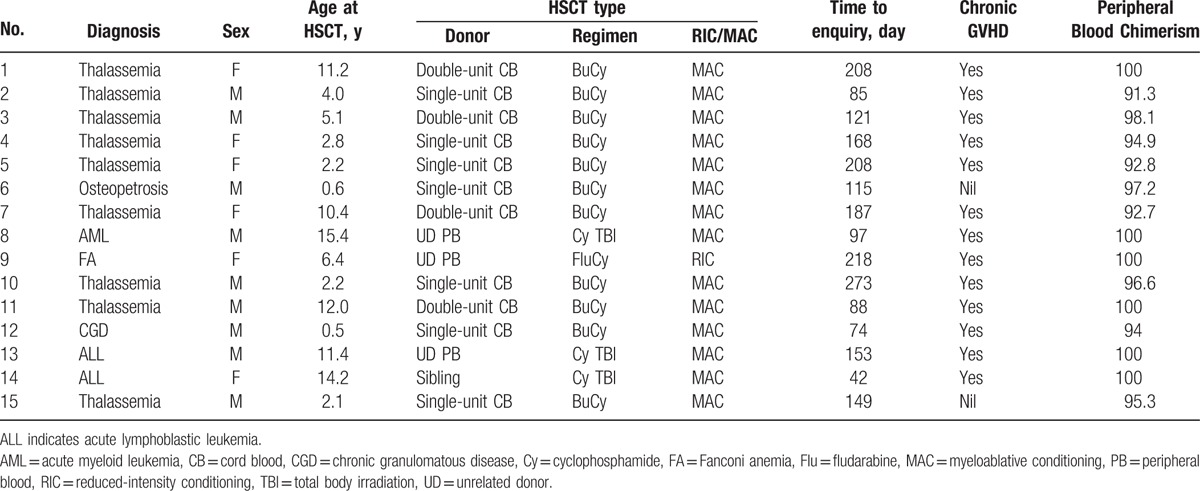
Clinical characteristics of patients with AIHA.

The median age was 5.1 years (range 0.5–15.4) at the time of HSCT and all but one had received unrelated donor transplant (12 umbilical cord blood, 3 adult matched unrelated donors). Six of these patients had concomitant thrombocytopenia. One patient had neutropenia at diagnosis. All patients require treatment with multiple agents including steroids for AIHA, with 12 of 15 (80%) patients achieving complete resolution of AIHA. Nine of the 15 patients had received rituximab in addition to steroid therapy. In 2 patients with steroid refractory AIHA, their parents were reluctant to use rituximab. No splenectomy was performed on these patients.

As shown in Table 3, 10 of 15 patients had a positive DAT with IgG polyspecific autoantibodies. Six patients also had complement fixation noted on red cells by direct antiglobulin testing. On eluate testing, the specificity of the antibody was identified as anti-e in 1 patient. Of these, 6 patients had major ABO incompatibility, 2 minor, and 3 major plus minor.

**Table 3:**
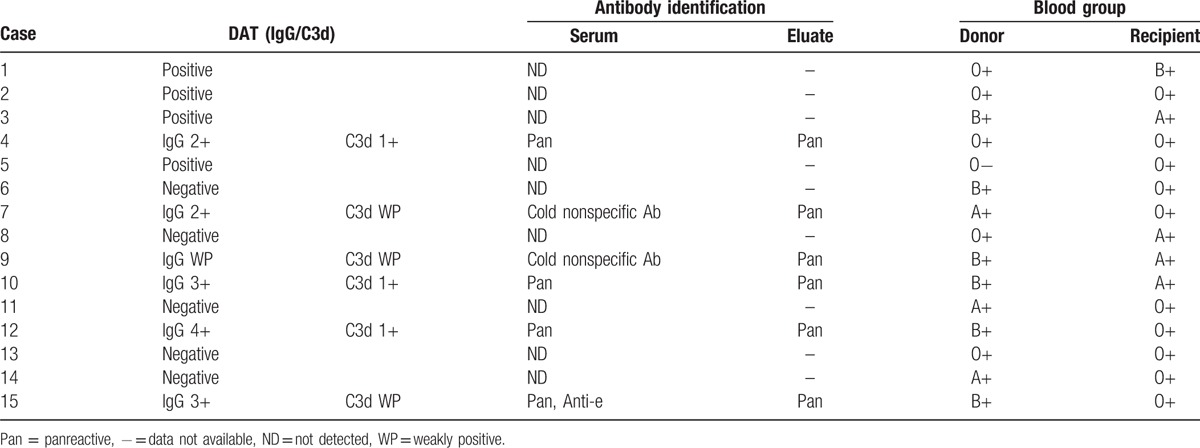
Results of DAT, antibody specificities from the serum, and red cell elution studies and blood group of the donor and the recipient.

### 3.2 Treatment and outcome

All cases had clinically significant hemolysis and were treated initially with methylprednisolone (4 mg/kg/day) and intravenous immunoglobulin (1 g/kg/day for 2 days). Ten of 15 patients had 95% to 100% unfractionated peripheral blood donor chimerism. The majority of AIHA patients were treated with >2 therapeutic agents; 5 of 15 patients (33%) received 3-agent therapy, 5 (33%) received 4 agents, and 4 patients (27%) received 5 agents. Clinical course of the AIHA cases are summarized in Table 4.

**Table 4:**
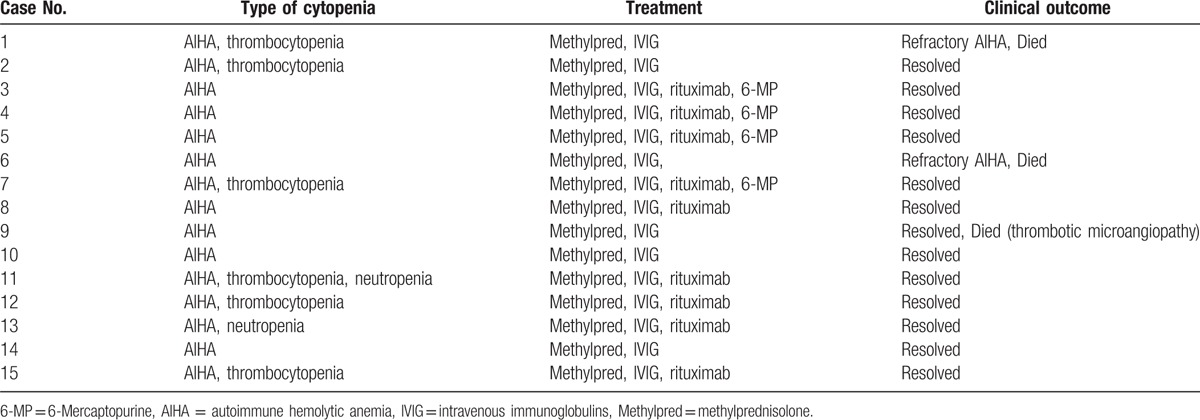
Clinical course for patients with AIHA.

Rituximab was readily available for the time period of study. In 9 patients, rituximab was subsequently added for persistent hemolysis despite first-line treatment. Methylprednisolone was tapered off within a month. Overall, the response to treatment was variable. However, after AIHA is addressed, thrombocytopenia may resolve on its own. Complete resolution of AIHA was achieved in 12 of 15 cases (80%). Refractory AIHA was observed in 2 of 15 cases (13%) and failed to control the hemolysis. One patient (7%) died of non-AIHA-related cause during AIHA treatment. As no other risk factor was identified in univariate analysis, multivariate analysis was not performed.

## 4 Discussion

AIHA has been reported anecdotally following allogeneic marrow transplantation, primarily in patients transplanted with T cell-depleted grafts.^[6]^ Development of AIHA after HSCT has been associated with younger recipients receiving cord blood transplantation (CBT) and concurrent chronic GVHD, suggesting a role for mismatched antigens in the pathogenesis of AIHA after allogeneic HSCT. As shown in Table 2, among the 15 patients with AIHA, 13 patients have developed chronic GVHD. This work reflected a similar trend to that reported by Page et al.^[10]^ Hemolysis and thrombocytopenia could also be a sign of microangiopathy.^[3]^ Hemolysis typically occurs from 2 to 25 months after HSCT with an incidence of 3.1% to 6%.^[11]^ Higher incidences of up to 15% to 20% have been reported,^[3]^ albeit in specific clinical settings and with less conventional conditioning regimens.

After allogeneic HSCT, all patients received cyclosporine to prevent GVHD and graft rejection, which is known to inhibit thymic-dependent clonal deletion and disrupt the reconstitution of the immune system.^[12]^ Among autoimmune conditions that develop after HSCT, AIHA is the most frequently reported.^[13,14]^ Despite advances in transfusion medicine, a simple immunohematological test such as DAT still remains the diagnostic hallmark of AIHA.^[7]^ However, manual DAT can only detect a level of 100 to 500 molecules of IgG/red cell and 400 to 1100 molecules of C3d/red cell. In other patients with negative DAT, but with clinical and hematological features typical of AIHA, IgA autoantibodies or monomeric IgM may be involved.^[15]^ However, transfusion should not be withheld in a critically ill patient even in the absence of compatible blood.

The most common treatment for β-thalassemia major involves lifelong regular blood transfusions. The development of anti-red cell antibodies can significantly complicate transfusion therapy.^[16]^ The development of anti-red cell antibodies can significantly complicate transfusion therapy. Red cell autoantibodies can result in AIHA, and in difficulty in cross-matching blood. The result indicated there is a statistically significant difference between patients with thalassemia major and in other conditions. The risk of alloimmunization is higher in patients who have received multiple blood transfusions such as thalassemia, and it may be possible to impact the incidence of AIHA following HSCT. Moreover, studies incorporating more in-depth analyses had demonstrated that 3% to 37% of multiply-transfused thalassemia patients possess antibodies against erythrocyte antigens.^[17–21]^ Alloimmune hemolytic anemia requires exposure to allogeneic red cells, the sources being pregnancy, blood product transfusion, and transplantation.

CD20 antibodies mediate their biologic effects against tumor cells through antibody-dependent cellular cytotoxicity, complement-dependent cytotoxicity, and direct apoptosis. Rituximab is a chimeric monoclonal antibody that responds specifically to the CD20 antigens found on the surface of malignant and normal B-cells.^[22,23]^ The majority of responders responded to rituximab within 4 weeks, whereas the rest responded more slowly, several weeks, or even months after rituximab treatment.^[24]^ Toxicity was manageable. It has been proposed that opsonized B cells with rituximab block macrophage Fc-receptor function, reducing platelets destruction in the spleen.^[25]^

This study has intrinsic limitations and pitfalls in the diagnosis, outcome, and patient population. First, it was difficult to accurately estimate the true number of patients with AIHA because of the retrospective nature of the study. It is possible that AIHA may have been missed in some patients because of mild symptoms or unremarkable laboratory results. For these reasons, the incidence of AIHA may have been underestimated. Second, the current study is a small retrospective study in a heterogeneous cohort. Some variables such as related versus unrelated donors and cord blood compared to PBSC are not statistically significant. Such reports based on small sample size will certainly require confirmation in larger studies, but they perhaps demonstrate the potentially important role. We conclude that AIHA after HSCT is a challenging endeavor. Therefore, conclusions drawn were representative only of those individuals who participated in the study and should be interpreted with caution and cohorts studies focusing on these patients should be undertaken.
